# A national facilitation project to improve primary palliative care: impact of the Gold Standards Framework on process and self-ratings of quality

**DOI:** 10.1136/qshc.2007.024836

**Published:** 2009-05-26

**Authors:** J Dale, M Petrova, D Munday, J Koistinen-Harris, R Lall, K Thomas

**Affiliations:** 1 Health Sciences Research Institute, Warwick Medical School, University of Warwick, Coventry, UK; 2 John Taylor Hospice, Birmingham, UK; 3 School of Health Sciences, University of Birmingham, Birmingham, UK

## Abstract

**Background:**

Improving quality of end-of-life care is a key driver of UK policy. The Gold Standards Framework (GSF) for Palliative Care aims to strengthen primary palliative care through facilitating implementation of systematic clinical and organisational processes.

**Objectives:**

To describe the general practices that participated in the GSF programme in 2003–5 and the changes in process and perception of quality that occurred in the year following entry into the programme, and to identify factors associated with the extent of change.

**Methods:**

Participating practices completed a questionnaire at baseline and another approximately 12 months later. Data were derived from categorical questions about the implementation of 35 organisational and clinical processes, and self-rated assessments of quality, associated with palliative care provision.

**Participants:**

1305 practices (total registered population almost 10 million). Follow-up questionnaire completed by 955 (73.2%) practices (after mean (SD) 12.8 (2.8) months; median 13 months).

**Findings:**

Mean increase in total number of processes implemented (maximum  = 35) was 9.6 (95% CI 9.0 to 10.2; p<0.001; baseline: 15.7 (SD 6.4), follow-up: 25.2 (SD 5.2)). Extent of change was largest for practices with low baseline scores. Aspects of process related to coordination and communication showed the greatest change. All dimensions of quality improved following GSF implementation; change was highest for the “quality of palliative care for cancer patients” and “confidence in assessing, recording and addressing the physical and psychosocial areas of patient care”.

**Conclusion:**

Implementation of the GSF seems to have resulted in substantial improvements in process and quality of palliative care. Further research is required of the extent to which this has enhanced care (physical, practical and psychological outcomes) for patients and carers.

The important role that primary care professionals play as providers of palliative care is widely recognised, but the processes, resources and skills to provide effective care are often lacking.^1^^–^5 In consequence, many patients in the UK lack access to well-coordinated, comprehensive community-based palliative care that can effectively address their psychosocial and physical needs. One specific consequence is that about 50% of patients do not die where they would choose, with stark disparity between those who wish to die at home and those who do so.

The Gold Standards Framework (GSF) for Palliative Care is an approach designed to strengthen the organisation and quality of primary palliative care (box 1). It is aimed at helping practices achieve key standards (the so-called 7 Cs) of care that are associated with quality outcomes (box 2).^6^ Its development was based on best available evidence of the clinical and organisational issues related to community palliative care. This included systematic reviews (including systematic reviews published by the Cochrane Pain, Palliative Care and Supportive Care Collaborative Review Group, 25 references), government and other guidelines in the UK and USA (5 references), searches for relevant publications in numerous publications databases (for 1990–2000, 301 references), registers of current research in the UK, USA and the European Union (17 references), and a systematic search of the “grey” literature from European sources (72 references). In addition, the themes that emerged were tested in focus groups with healthcare professionals.^6^ ^7^

**Box 1 Overview of the GSF^6^**

The GSF aims to facilitate primary healthcare teams to achieve:

- optimal control of patients’ symptoms;
- patients enabled to live and die well, in their preferred place of choice;
- fewer crises and unplanned admissions to hospital or hospice;
- better supported, informed and empowered patients and carers;
- greater confidence in primary healthcare teams in the care they provide;
- more effective teamworking;
- better communication between team members and patients/carers.

Following initial piloting with 12 general practices in 2001, the framework’s national rollout was sponsored by the charity Macmillan Cancer Support and more recently the NHS (England) End of Life Care Programme^7^ and, in Scotland, the National Lottery. The framework has now been adopted by around 4500 practices, covering almost half of the English population, and in Scotland, two-thirds of practices are involved, making it one of the largest primary care facilitation projects to have ever been undertaken.

Implementation of the GSF is facilitated by local facilitators who have been appointed by primary care organisations to develop local interest and introduce GSF to practices in their area. While some facilitators are funded or seconded to work two to four sessions every week to implement the GSF, others have been funded full time for a fixed period. GSF facilitators come from many different backgrounds, but the majority are general practitioners (GPs) or district nurses, clinical nurse specialists or project managers. Facilitators support the GSF practices by organising local GSF practice coordinator meetings, the registration of practices and collection of GSF questionnaires.

A national GSF team supports facilitators through workshops, an advice line, a newsletter and website resources including a password-protected facilitator support section. Practices have access to a website (http://www.goldstandardsframework.nhs.uk) with practical tools, guidance documents and examples of good practice. These integrate many established aspects of primary palliative care: identifying palliative patients systematically, naming a lead GP and community nurse for each patient, coordinating multidisciplinary working through regular meetings, advance planning of care with patients, etc.

**Box 2 Aspects of the GSF programme: the seven Cs^6^**

**C1—Communication**: Palliative care register maintained; regular primary healthcare team (PHCT) meetings held to discuss and plan care for patients on the register.

**C2—Coordination**: A practice coordinator (eg, GP, district nurse or manager) oversees the work of GSF, acting as a contact for the local GSF facilitator; record maintained of preferred place of death; named GP and district nurse for each patient.

**C3—Control of symptoms**: Effective holistic management of all aspects of care, including the use of physical assessment tools to identify and address physical symptoms and psychosocial and spiritual needs.

**C4—Continuity**: A key PHCT member identified for each patient and information flow maintained, eg, with out-of-hours providers.

**C5—Continued learning**: Practice-based education in palliative care, including audits and significant event analysis

**C6—Carer support**: Identifying and meeting the needs of informal carers, before and after the patient’s death

**C7—Care of the dying**: Identifying terminal phase, managing it appropriately, using protocols such as the Liverpool Care Pathway; availability of anticipatory care procedures; assessing need for continued medication in last days of life; informing family when dying phase is approaching.

The GSF’s evolution has been remarkable, with dramatic uptake and incorporation into UK health policy. Evaluation has been intrinsic at every stage, and several local healthcare audits have been undertaken, but hitherto there has been only limited published evidence of its impact. Even so, it has been recognised as an effective programme by the National Institute for Health and Clinical Excellence (NICE) in 2004, endorsed by the Royal College of General Practitioners in 2005 and identified in the 2006 Government White Paper on community services as a central aspect of health policy.^8^^–^10 The GSF is intended to be applicable to end-of-life care in general, not just cancer care, and its key elements can be applied in any healthcare setting.

A small initial pilot study by KT of early adopting practices found that palliative care patients were more systematically identified and that communication about them had improved following implementation of GSF.^7^ Despite wide variation in how teams implemented the programme, with different levels of commitment amongst professionals within individual practices, one consequence appeared to be increased consistency of care with “fewer people slipping through the net”.^11^ This variation in implementation has been confirmed more recently.^12^

The current study was undertaken as part of a national evaluation of phases 3–6 of the GSF programme in England and Northern Ireland; phases 1 and 2 had been extended pilots.^10^ ^11^ The aims of the evaluation were:

- to describe the practices that participated;
- to describe changes that occurred in the year following entry to the programme in practices’ processes associated with the provision of palliative care;
- to identify factors associated with the extent of change that occurred.

## METHODS

### Setting and recruitment

General practices in England and Northern Ireland were recruited through written and, when possible, electronic promotional materials sent to the service improvement leads and nurse directors of each cancer network, members of the Macmillan GP network (approximately 100 GPs), existing GSF area facilitators, and the primary care cancer leads of primary care trusts (PCTs). Most practices received no financial reward for participating in the GSF programme; a small minority of PCTs in England incentivised participation through enhanced local delivery plans.

Interested practices were contacted by local palliative care facilitators. These tended to be GPs with a special interest in palliative care, district nurses or palliative care nurses, and were generally funded by the local PCT or cancer network. In all, there were approximately 190 facilitators involved in the local implementation of the programme between 2003 and 2005.

### Data collection

Data on each participating practice were collected through self-completed questionnaires at baseline (as part of their registration with the programme), and again 6–12 months later. Questionnaires were mailed to the GP or nurse named by the practice as its coordinator of GSF implementation, with a covering letter. Approximately 4 weeks later, non-responding practices were sent a second copy of the questionnaire, and 3 weeks after this, reminder phone calls were made.

For GSF phases 3–5, practices completed follow-up questionnaires at 6 and 12 months. As there were very few differences between the responses given at 6 and 12 months, phase 6 practices were asked to complete only one follow-up questionnaire at 12 months. The follow-up questionnaires used in the analysis were the latest questionnaire that each practice completed.

The questionnaire was based on the original one used in GSF phases 1 and 2, developed by KT with palliative care academics based on a systematic review of the literature, and modified by a team of researchers and clinicians following initial piloting. It contains 62 questions about practices’ provision of palliative care and use of the GSF, of which 41 were analysed for the purposes of the current study: 35 tick-box *process* questions (predominantly yes/no) addressing issues of process and organisational structure, such as whether the practice holds regular team meetings or has a register for palliative care cancer patients; and 6 Likert-type *rating* questions (each with a scale of 1 “very poor” to 5 “very good”) about aspects of quality, such as teamwork in the practice.

### Data analysis

The data were summarised using descriptive statistics (mean (SD)) to describe the degree of implementation of each process reported on in the questionnaires at baseline and follow-up and describe the extent of change. A total *process* score was calculated at baseline and follow-up by summating the responses over all the process scores (range of total score: 1 to 35, with 1 indicating the lowest level of implementation of processes). Absolute change was calculated by subtracting baseline from follow-up scores. The difference in the scores was not normally distributed and therefore the Wilcoxon signed ranked test was used to assess whether the difference was statistically significant.

Several variables were identified as potential predictors of baseline total score, the final total score and the extent of change. Linear regression models using forward stepwise method were used to select variables. In this approach, each variable was added to the model sequentially. At each step, each variable that was not already in the model was tested for inclusion in the model and was added to the model, provided that its p value was below the preset level of 0.05. The final model was based on a set of predictors that were selected using this process, and it provided the adjusted means (together with the 95% CI). Only practices that completed all items in both questionnaires were included in this modelling. The computations were done using SAS V9.1.3. The independent variables included in the model were practice characteristics (type of geographic location, cancer network, training status and size of registered population), GSF phase and responses to the six rating questions at baseline and follow-up.

Descriptive statistics were used to describe the responses to the rating questions at baseline and follow-up. In addition, absolute change for each item (final rating minus baseline rating) was calculated, and responses to the rating questions at baseline and final were analysed in relation to process scores in terms of differences in patterns of response to the rating questions between low-scoring and high-scoring practices.

## FINDINGS

In total, 1837 practices expressed interest in participation, of which 1455 (79.2%) registered for participation. Of these, 1305 (89.7%) completed a baseline questionnaire. Practices were located across all regions of England and Northern Ireland, and had a registered population of almost 10 million people (ie, about 15% of the total population). Table 1 shows the characteristics of the 1305 practices that participated and the subgroup of 955 (73.2%) that submitted at least one follow-up questionnaire. For the latter, the period between completion of the baseline and final follow-up questionnaire was a mean (SD) of 12.8 (2.8) months (median 13 months).

**Table 1:** Characteristics of the participating practices

	Practices returning baseline questionnaire (n = 1305)	Practices returning baseline and follow-up questionnaires (n = 955)
Practice size, patients		
1–5000	372 (28.5%)	269 (28.2%)
5001–10 000	588 (45.1%)	426 (44.6%)
10 001–15 000	262 (20.1%)	202 (21.2%)
15 001–20 000	39 (3.0%)	31 (3.2%)
20 001–25 000	9 (0.7%)	5 (0.5%)
25 001+	4 (0.3%)	4 (0.4%)
Location	626 (48.0%)	439 (46.0%)
Urban		
Semi-rural and mixed	414 (31.7%)	305 (32.0%)
Rural	200 (15.3%)	160 (16.8%)
Training practice	533 (40.8%)	399 (41.8%)
No of registered patients	9 830 000	7 390 000

### Missing data

Of the 955 practices that provided follow-up data, the non-response rate to individual process questions varied from 0.4% to 6.0% at baseline, and 0.4% to 4.2% at follow-up; for individual rating questions it varied from 1.4% to 2.7% and 1.2% to 2.2% respectively. The cumulative impact of non-response to individual questions resulted in only 505 (52.9%) practices providing responses to all process questions and only 449 (47.0%) responding to all process and rating questions together with complete demographic information in both questionnaires. However, the practice characteristics and pattern of responses from the 505 and 449 practice datasets were very similar to those of all 955 practices: two-sample t tests showed no significant differences between the 449, 505 and 955 datasets in total process scores at baseline and final, or in the degree of change. Mann–Whitney tests showed no significant difference between the three datasets in the ratings of quality at baseline and final.

### Change in process and rating scores

**Figure 1:**
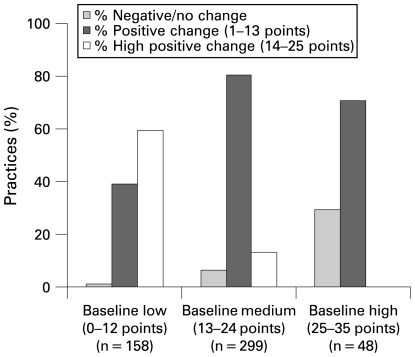
Degree of change relative to baseline process score.

Table 2 shows the level of implementation of processes related to palliative care that practices reported at baseline and follow-up. The mean (SD) for the total number of processes implemented (maximum possible  = 35) increased by 9.6 (95% CI 9.0 to 10.2; p<0.001), from 15.7 (6.4) at baseline to 25.2 (5.2) at follow-up. Change was largest for practices with low baseline scores (total score <13 points); these achieved a mean change of 14.8 points (95% CI 13.9 to 15.7; interquartile range 11–19 points). Those with medium scores at baseline (13–24 points) had a mean change of 8.2 points (95% CI 7.7 to 8.8; interquartile range 5–11), while high-scoring practices (>25 points) achieved a mean change of only 1.2 points (95% CI –0.4 to 2.9; interquartile range 0–4.8). Figure 1 illustrates the pattern of change relative to baseline process score.

**Table 2:** Responses to process questions at baseline and follow-up

Process question	Affirmative answer (%)		
	All practices returning baseline questionnaire (n = 1305)	Practices returning baseline and final questionnaires (n = 955)	
	Baseline	Baseline	Final
1. Up-to-date cancer register	723 (55.4)	515 (53.9)	889 (93.1)
2. Up-to-date palliative care register	290 (22.2)	219 (22.9)	852 (89.2)
3. Regular primary healthcare team (PHCT) meetings	865 (66.3)	648 (67.9)	769 (80.5)
4. Regularly discuss palliative care patients at PHCT meeting	339 (26.0)	256 (26.8)	735 (77.0)
5. Use a palliative care communication tool	906 (69.4)	675 (70.7)	878 (91.9)
6. Regular planned practice meetings with palliative care specialist(s)	549 (42.1)	412 (43.1)	565 (59.2)
7. Nominated coordinator within practice for palliative care	582 (44.6)	441 (46.2)	871 (91.2)
8. Inform secondary care specialist of coordinator’s details	135 (10.3)	102 (10.7)	337 (35.3)
9. Record advanced care planning with whole PHCT	279 (21.4)	221 (23.1)	657 (68.8)
10. Record advanced care planning with patients/carers	689 (52.8)	528 (55.3)	835 (87.4)
11. Record preferred place of care/death	313 (24.0)	239 (25.0)	681 (71.3)
12. Note lead general practitioner and district nurse for each patient	562 (43.1)	437 (45.8)	786 (82.3)
13. Use physical symptom assessment tool for palliative care needs	386 (29.6)	294 (30.8)	421 (44.1)
14. Routinely assess palliative care patients’ physical needs and symptom control	1072 (82.1)	785 (82.2)	910 (95.3)
15. Routinely assess psychosocial needs	928 (71.1)	689 (72.1)	859 (89.9)
16. Routinely assess religious/spiritual needs	482 (36.9)	366 (38.3)	536 (56.1)
17. System/protocol to routinely assess patients’ expressed needs	551 (42.2)	414 (43.4)	721 (75.5)
18. Send handover form to out-of-hours care provider	547 (41.9)	414 (43.4)	786 (82.3)
19. Practice-based educational palliative care events in last 6 months	409 (31.3)	305 (31.9)	337 (35.3)
20. Palliative care review/audit meeting in last 6 months	224 (17.2)	166 (17.4)	487 (51.0)
21. Regularly use palliative care significant event analysis	786 (60.2)	582 (60.9)	721 (75.5)
22. Maintain carers’ database	396 (30.3)	292 (30.6)	476 (49.8)
23. Offer specific leaflets/information to carers	530 (40.6)	402 (42.1)	665 (69.6)
24. Give carers information on what to do after death	801 (61.4)	609 (63.8)	778 (81.5)
25. Routinely assess main carer’s practical needs	855 (65.5)	643 (67.3)	807 (84.5)
26. Routinely assess main carer’s psychosocial needs	596 (45.7)	455 (47.6)	653 (68.4)
27. Give carers information regarding statutory services	557 (42.7)	407 (42.6)	633 (66.3)
28. Have a practice protocol for care of the bereaved	272 (20.8)	201 (21.0)	376 (39.4)
29. Have practice protocol for care of dying cancer patients	205 (15.7)	160 (16.8)	439 (46.0)
30. Follow a care pathway	166 (12.7)	120 (12.6)	362 (37.9)
31. Have a procedure for use of anticipatory medication in the home	633 (48.5)	467 (48.9)	786 (82.3)
32. Routinely assess and discontinue inappropriate medication	1081 (82.8)	807 (84.5)	906 (94.9)
33. Inform family when cancer patient is entering the dying phase	1213 (93.0)	896 (93.8)	930 (97.4)
34. Extended principles to non-cancer patients	653 (50.0)	486 (50.9)	662 (69.3)
35. Extended work to patients from point of diagnosis	374 (28.7)	270 (28.3)	428 (44.8)

The aspects of practice that showed the greatest level of change were related to coordination and communication, both being processes for which there was considerable scope for improvement at baseline. For example, at follow-up, 90.2% of practices described having a register of patients in need of palliative care compared with 23.4% at baseline, 78.9% (27.0% at baseline) regularly discussed palliative cancer patients at team meetings, 70.6% (23.4% at baseline) recorded advance care planning with the PHCT, and 72.8% (25.4% at baseline) recorded palliative care patients’ preferred place of death (p<0.001 for all comparisons). For some processes the gain was relatively modest. For example, at follow-up only a minority of practices reported the use of a physical symptom assessment tool or holding practice-based educational events on cancer/palliative care.

Practices generally rated the quality of their palliative care as being at least “good” on each of the six dimensions at baseline (with the exception of “quality of support offered to carers” which had a median rating of “average”), and across all dimensions self-ratings improved in the 12 months following GSF implementation (for each item p<0.001) (table 3). The degree of change was highest for ratings of the “quality of palliative care for cancer patients” and “confidence in assessing, recording and addressing the physical and psychosocial areas of patient care”, and least for “quality of support to staff” and “quality of teamwork in practice”. However, as indicated by the ranges shown in table 3, there were some practices for which change occurred in a negative direction.

**Table 3:** Responses to rating questions at baseline and follow-up* and the difference between scores

Quality measure addressed		No of practices	Range (minimum– maximum)	Mean (SD)	Median	Wilcoxon signed ranks test statistics on significance of the difference between baseline and final ratings
Confidence regarding physical/psychosocial patient care	Baseline	937	1–5	3.51 (0.77)	4.00	
	Final	942	1–5	3.94 (0.67)	4.00	
	Difference	924	−3–3	0.43 (0.86)	0.00	Z = −13.44; p<0.001
Quality of support offered to carers	Baseline	936	1–5	3.38 (0.78)	3.00	
	Final	944	2–5	3.80 (0.69)	4.00	
	Difference	926	−2–4	0.42 (0.88)	0.00	Z = −13.10; p<0.001
Quality of support offered to practice staff	Baseline	929	1–5	3.48 (0.82)	4.00	
	Final	934	1–5	3.81 (0.73)	4.00	
	Difference	909	−3–4	0.32 (0.89)	0.00	Z = −10.11; p<0.001
Quality of teamwork in the practice	Baseline	942	1–5	3.83 (0.81)	4.00	
	Final	943	1–5	4.16 (0.73)	4.00	
	Difference	931	−3–3	0.32 (0.89)	0.00	Z = −10.34; p<0.001
Quality of palliative care for cancer patients	Baseline	935	1–5	3.72 (0.73)	4.00	
	Final	936	1–5	4.16 (0.63)	4.00	
	Difference	916	−3–4	0.44 (0.81)	0.00	Z = −14.40; p<0.001
Level of co-working with palliative care specialists	Baseline	933	1–5	3.65 (0.83)	4.00	
	Final	936	1–5	4.07 (0.72)	4.00	
	Difference	914	−2–4	0.41 (0.92)	0.00	Z = −12.47; p<0.001

*Range: 1 “very poor” to 5 “very good”.

### Predictors of baseline and follow-up process and quality rating scores

For the 449 practices that completed all parts of the questionnaire and included in the generalised linear model, no significant associations were identified between practice characteristics or GSF phase and total process scores. Responses to three of the rating questions at baseline (“confidence in assessing, recording and addressing the physical and psychosocial areas of patient care”; “quality of support offered to carers”; and “quality of palliative care for cancer patients”) were associated with total baseline process score (p<0.001 for each measure). For example, for practices that rated the quality of palliative care they provided as “average”, the adjusted mean of the total process score at baseline was 14.2 (95% CI 12.9 to 15.4), compared with 17.2 (95% CI 16.0 to 19.4) for those that rated their care as “very good”.

The extent of change that occurred in process scores was directly associated with the rating of the quality of palliative care at follow-up and inversely with the rating of support to carers at baseline (p<0.001). For example, practices that rated their quality of palliative care at follow-up as “very good” had an adjusted mean change of 11.6 (95% CI 10.8 to 12.4), while for those that rated their care as ‘average’ it was only 4.3 (95% CI 2.9 to 5.6). On the other hand, for practices that rated the support they offered to carers at baseline as “poor”, the adjusted mean change was 10.3 (95% CI 8.9 to 11.6), while for practices that rated it as “very good”, it was 6.9 (95% CI 5.1 to 8.6). This suggests that the extent to which practices are dissatisfied with their provision of support to carers affects the motivation to improve palliative care processes.

Three variables emerged as being strongly associated with *final* total process score. These were baseline total process score, and final responses to the rating questions about “confidence in assessing, recording and addressing the physical and psychosocial areas of patient care” and “quality of palliative care for cancer patients” (p<0.001 for each item). The more positive the response to each of these rating questions, the higher the adjusted mean final total score. For example, practices rating their confidence as “very good” had an adjusted mean final total score of 26.6 (95% CI 25.4 to 27.7), whereas those that rated it as “poor” had an adjusted mean of 15.1 (95% CI 11.5 to 18.7).

## DISCUSSION

The findings from this study suggest that the GSF in community palliative care offers a helpful means of facilitating the uptake of processes associated with high-quality care. Despite a general lack of financial incentive for participation at the time of this study, the GSF achieved a very high level of uptake. Over 1300 practices entered the scheme, with registered populations covering about 15% of the population of England and Northern Ireland. The vast majority reported improvement in their processes related to palliative care and procedures and their perceptions of the quality of care provided.

The implementation of palliative care processes by practices was *not* associated with their size, training status, location, nor the phase of the programme that they entered. This implies that other aspects, such as the attitude, motivation and team dynamics within a practice, may have been more important in supporting or hindering adoption of new processes rather than local health priorities or the specific health policy context (which underwent considerable change between 2003 and 2005).^12^ Although practices that were already high scoring at baseline showed least improvement in their process scores, it is possible that such practices made use of the GSF to improve their existing processes and thereby the quality of their palliative care in ways that the audit questionnaire could not capture.

One of the most remarkable changes that practices reported having made was the introduction of a palliative care register: 23.4% had this when they entered the programme compared with 90.2% a year later. Although evidence is lacking that the use of a register leads to more effective outcomes in palliative care, registers in other areas of healthcare have been found to lead to improved practice. For example, in chronic disease management in general practice, registers have been shown to support the achievement of high-quality care.^13^ However, the relationship between process and outcome is complex and likely to be mediated by a host of structural and organisational issues.

Practices that rated highly the quality of their palliative care were more likely than other practices to have formal processes in place to support such care, which suggests that practices perceive an association between primary palliative care process and outcome. In addition, there was evidence that practices that had the greatest self-confidence in the quality of palliative care they provided were those that introduced the largest number of new processes, and had so experienced the greatest impact from participating in the GSF programme.

### Methodological considerations

A number of methodological issues need to be considered in the interpretation of this finding. First, although the GSF has had very high uptake across the UK, it is likely that practices that volunteered to participate had a greater interest in, and were more motivated to improve, the quality of primary palliative care than practices that did not. This level of interest probably contributed to the very high questionnaire response rates, and hence the representativeness of findings. Their generalisability to practices that chose not to participate in the GSF programme cannot be assumed.

Second, the data for this evaluation were self-reported, and responses may have reflected the view of a single respondent rather than the collective view of a practice. Sometimes different respondents completed baseline and follow-up questionnaires. The reliability and validity of self-completed questionnaires when completed by health professionals is an area of quite limited research. While self-reports allow access to large amounts of data at relatively minimal cost,^14^ there are several limitations: answers can be incorrect due to lack of understanding or (room for) explanation^15^; forgetting the necessary information^16^; giving an intentional/unintentional false answer^17^; and a tendency to omit, withhold or minimise data if they are not socially desirable and to answer “yes” to questions.^18^ ^19^ Memory recall may have considerable impact,^16^ and the frequency that processes are used will influence how accurately they are recalled.

Third, no attempt was made to weight the importance of individual processes or the complexity of implementing them in assessing the degree of change that occurred between baseline and follow-up. This aspect of the questionnaire could be developed through further research.

Finally, it was beyond the scope of this evaluation to evaluate the extent to which individual practices made use of the GSF resources made available to them or how this affected the degree of change that they reported or the degree to which patients benefited from the changes. However, we have previously reported a qualitative case study of 15 practices, selected to reflect the diversity of practices participating in GSF, conducted up to 2 years after the GSF follow-up questionnaire had been completed.^12^ This found a broad level of consistency between the GSF process data scores (as described above) and the quality of palliative care activity observed within practices, particularly for practices with high questionnaire scores. This finding provides support for both the validity of the GSF questionnaire as well as the sustainability of change that occurs. Supportive team relationships emerged as critically influencing effective and sustainable palliative care activity within practices. Marginal performance, by contrast, occurred where commitment was less pervasive, practice priorities conflicted, and/or where there was changing personnel. Conducting trials to assess the clinical effectiveness of complex interventions in palliative care is acknowledged as problematic^20^ and new methods are needed to enable patient outcomes to be evaluated. A current study is assessing the feasibility of comparing care given to patients in practices before taking up GSF and following its implementation.^18^ 

## CONCLUSIONS

The GSF is unique for its scale and focus on a particularly challenging aspect of care that requires high level organisational and clinical processes across all hours of the day and week. Between 2003 and 2005, the uptake of the GSF was impressive and the findings from this study provide evidence of the degree of change that occurred following implementation. To what extent change across practices has led to enhanced quality of palliative care, in terms of physical and psychological outcomes for patients and improved carers’ and health professionals’ experience of care, requires further research to elucidate the relationship between GSF-recommended processes and outcomes. Processes, such as palliative care registers and primary care team meetings, may be sophisticated or superficial, but it is the way that they are used that is likely to determine their influence on the quality of care received by patients. This suggests a need for developing more sensitive measures of quality, both for primary palliative care as well as for other long-term conditions where the need for strong process in the context of effective organisational relationships is likely to be fundamental to achieving quality care.^21^ ^22^
